# Low hemoglobin increases risk for cerebrovascular disease, kidney disease, pulmonary vasculopathy, and mortality in sickle cell disease: A systematic literature review and meta-analysis

**DOI:** 10.1371/journal.pone.0229959

**Published:** 2020-04-03

**Authors:** Kenneth I. Ataga, Victor R. Gordeuk, Irene Agodoa, Jennifer A. Colby, Kimberly Gittings, Isabel E. Allen

**Affiliations:** 1 University of Tennessee Health Science Center, Memphis, TN, United States of America; 2 University of Illinois at Chicago College of Medicine, Chicago, IL, United States of America; 3 GBT, South San Francisco, CA, United States of America; 4 Xcenda LLC, Palm Harbor, FL, United States of America; 5 School of Medicine, University of California, San Francisco, San Francisco, CA, United States of America; Université Claude Bernard Lyon 1, FRANCE

## Abstract

Sickle cell disease (SCD) is characterized by deoxygenation–induced polymerization of hemoglobin in red blood cells, leading to hemolytic anemia, vaso–occlusion, and the development of multiple clinical complications. To characterize the clinical burden associated with differences in hemoglobin concentration and hemolysis measures, a systematic literature review of MEDLINE, EMBASE, and related meta–analyses was undertaken. For quantitative analyses related to hemoglobin concentration, pooled results were analyzed using random effects models to control for within–and between–study variability. To derive risk ratios associated with hemoglobin concentration change, we combined ratios of means from select studies, which reported hazard and odds ratios in meta–analyses for hemoglobin concentration–related outcomes and changes between groups. Forty-one studies were identified for inclusion based on relating hemoglobin concentration to clinical outcomes. Meta–analyses demonstrated that mean hemoglobin concentration was significantly lower in patients with cerebrovascular disease (0.4 g/dL), increased transcranial Doppler velocity in cerebral arteries (0.6 g/dL), albuminuria (0.6 g/dL), elevated estimated pulmonary artery systolic pressure (0.9 g/dL), and in patients that subsequently died (0.6 g/dL). In a risk reduction meta–analysis, modeled increased hemoglobin concentrations of 1 g/dL or greater resulted in decreased risk of negative clinical outcomes of 41% to 64%. In conclusion, chronic anemia is associated with worse clinical outcomes in individuals with SCD and even modest increases in hemoglobin concentration may be beneficial in this patient population. This systematic review has been registered on Prospero (Registration number CRD42018096860; https://www.crd.york.ac.uk/prospero/).

## Introduction

Sickle cell disease (SCD) is characterized by deoxygenation–induced polymerization of hemoglobin in red blood cells (RBCs), leading to altered blood rheology, hemolysis and vaso–occlusion. Hemoglobin concentration is one indicator of the degree of hemolysis that occurs in SCD, as evidenced by the strong inverse correlation of hemoglobin concentration with clinical measures of RBC destruction, such as reticulocyte count and serum concentrations of lactate dehydrogenase, indirect bilirubin, and aspartate transaminase, and by the direct correlation of hemoglobin concentration with serum haptoglobin concentration [1, 2]. The chronic hemolytic anemia experienced in varying degrees by patients with SCD leads to reduced oxygen–carrying capacity, tissue hypoxia, and clinical manifestations such as fatigue. Acute worsening of anemia may occur for several reasons, including increased hemolysis related to vaso–occlusive episodes, acute splenic sequestration, transient red cell aplasia, and hyperhemolysis following transfusion reactions [3]. Episodes of acute illness and chronic complications in SCD lead to a decreased quality of life, making SCD one of the most clinically severe monogenic disorders worldwide [4, 5].

Along with chronic hemolysis, vaso–occlusion, and tissue ischemia, SCD is characterized by progressive end–organ damage of the heart, brain, lungs, spleen, liver, kidneys, and bones [6, 7]. Although most patients with SCD in resource–rich countries live beyond childhood, the median life expectancy remains low, and is reduced by 2 to 3 decades [8]. This increased risk of early mortality is, in large part, due to the development of multiple end–organ damage [9–12].

Individual studies have correlated low hemoglobin concentration with poor patient outcomes. Hemoglobin concentrations less than 8 g/dL are associated with complications during hospitalization, silent cerebral infarcts, and mortality [13–15]. Decreases in hemoglobin concentration of 1 g/dL are associated with approximately two–fold greater odds of microalbuminuria [16]. To comprehensively quantify the clinical burden associated with low hemoglobin concentration in patients with SCD, we conducted a systematic literature review and meta–analysis. As a secondary analysis, we conducted a meta–analysis of risk ratios associated with hemoglobin change across selected clinical endpoints including mortality in SCD.

## Materials and methods

Systematic literature searches of Excerpta Medica (EMBASE) and MEDLINE (via PubMed) databases were conducted (January 1, 1998 to February 26, 2019) to identify studies reporting on associations of hemoglobin concentration and other measures of hemolysis with clinical outcomes in patients with SCD. Key search terms included “sickle cell anemia”, “sickle cell disease”, “hemoglobin”, “haemoglobin”, “hemolysis”, and “haemolysis” (see S1 Appendix of S1 and S2 Tables for full search strategies). In addition to the EMBASE and MEDLINE databases, abstracts from the previous 2 years of 5 prominent scientific conferences, where results of studies in SCD are presented (American Society of Hematology; European Hematology Association; Annual Sickle Cell Disease Research and Educational Symposium and National Sickle Cell Disease Scientific Meeting; International Society for Blood Transfusion; American Society of Pediatric Hematology/Oncology), were evaluated to identify relevant literature yet unpublished in manuscript form.

Citations identified from the database and grey literature searches were reviewed in a 2–step process by a single reviewer: first pass title/abstract evaluation followed by full text assessment of selected papers. Studies were screened for English language publications reporting on a population of pediatric or adult patients with SCD. Outcomes of interest were the associations of clinical complications with hemoglobin concentration and selected measures of hemolysis: reticulocyte count (percentage and absolute counts); unconjugated or indirect bilirubin; serum lactate dehydrogenase; dense RBCs; plasma cell–free hemoglobin concentration; and urine hemoglobin. Clinical outcomes included efficacy, safety, acute and chronic clinical complications, and mortality. In an effort to report on a robust body of evidence, the current analysis focuses on hemoglobin concentration and 4 key clinical outcomes, namely stroke and silent cerebral infarction, albuminuria (defined as an albumin–creatinine ratio of ≥ 30 mg/g; moderately increased albuminuria: an albumin–creatinine ratio 30–299 mg/g), elevated estimated pulmonary artery systolic pressure (PASP; based on tricuspid regurgitant jet velocity [TRV] ≥ 2.5 m/s), and mortality. Included study designs were phase 2, 3, or 4 randomized controlled trials (RCTs), open-label trials, single-arm trials, prospective or retrospective observational studies, database studies, registry studies, or surveys. Phase 1 clinical trials, pharmacokinetic/pharmacodynamic studies, preclinical or animal studies, case studies, reports or series, guidelines, editorials, letters, commentaries, narrative literature reviews, and dissertations were excluded. All sickle cell disease genotypes were included and studies were included regardless of hydroxyurea or transfusion therapy use.

### Data analysis

Data extraction was performed (JAC; KG) according to a predetermined format of study characteristics and outcomes data. Key elements included study characteristics, patient demographics, and measures of hemoglobin concentration associated with clinical outcomes of interest. Data were extracted by a single reviewer with select validation by a second reviewer. Quantitative analyses were conducted specifically for hemoglobin as the key outcome of interest. The study findings were separated into categories of outcomes related to hemoglobin levels. The differences in the hemoglobin concentration for patients with and without each outcome (i.e., mortality, albuminuria, etc.) were analyzed. For each category, study findings were aggregated to perform separate meta–analyses assessing the overall magnitude of the association. Findings were stratified by levels of hemoglobin concentration to examine heterogeneity. Heterogeneity was measured using I^2^ values and Cochran’s Q statistic. Pooled results were analyzed using random effects models to control for within and between study variability.

Sensitivity analyses were performed to examine potential publication bias, including jackknife analyses and Begg and Egger statistics [17]. These findings have been reported in the results in addition to the primary study findings and subgroup analyses [18]. Furthermore, meta–regression was performed to understand how study traits contributed to heterogeneity of pooled effect estimates [19]. The meta–analytic results are displayed using forest plots [20].

For the meta–analysis evaluating risk ratios associated with change in hemoglobin concentration, ratios of means from select studies and reported hazard and odds ratios in meta–analyses by change in hemoglobin concentration between groups for each outcome were combined [21]. When studies reported separate values for hemoglobin concentration outcomes in bivariate and multivariate analyses, these were combined within the study prior to the overall meta–analysis for studies in an outcome category. Analyses were performed using STATA 13.2 (Stata, College Station, TX, USA).

Three quality assessment scales were utilized to evaluate included studies: (1) the Cochrane Collaboration tool for assessing the risk of bias in RCTs; (2) the Newcastle–Ottawa Scale for evaluation of quality in prospective, non–randomized studies [22], and (3) the checklist developed by the International Society for Pharmacoeconomics and Outcomes Research for evaluating retrospective databases and registries [23]. Quality assessment was completed by two researchers independently, with discrepancies in scoring discussed with a third researcher to determine the final assessment. Results of the quality assessments may be found in the Appendix.

This systematic review has been registered on Prospero (Registration number CRD42018096860; https://www.crd.york.ac.uk/prospero/).

## Results

**Fig 1:**
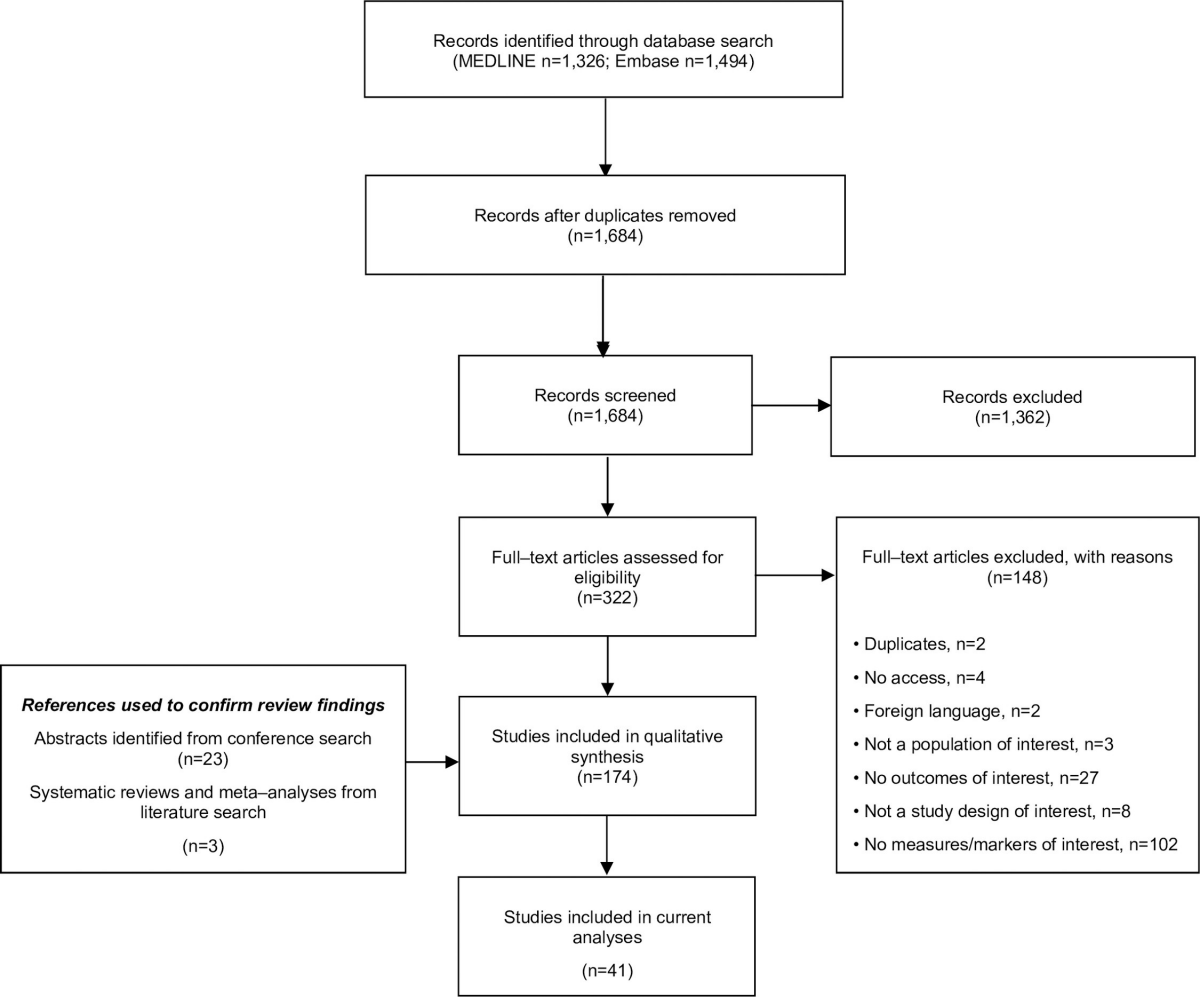
PRISMA diagram for the literature selection and review process.

Initial results of the database searches returned a total of 2,820 potential publications (Fig 1). Following the removal of duplicate citations and a review of the titles and abstracts, 322 publications remained for evaluation. Review of the full text manuscripts resulted in the inclusion of 174 publications for qualitative synthesis. Of these analyses, 41 reported on the direct relationship between hemoglobin concentration and the key clinical outcomes of interest: stroke and silent cerebral infarct, albuminuria, elevated estimated PASP, and mortality. Key study characteristics are provided in Table 1.

**Table 1:** Included studies.

First Author, Year	Study Design	Study Country	Number of Included Patients	Mean Patient Age (years)	Included Outcomes
Al-Allawi, 2016 [24]	Prospective cohort	Iraq	94	≥3	Elevated TRV
Aleem, 2010 [25]	Prospective cohort	Saudi Arabia	67	23.8	Proteinuria
Ambrusko, 2006 [26]	Retrospective	US	44	14.82	TRV elevation
Ataga, 2006 [27]	Prospective cohort	US	76	38.4 vs. 42.3	Risk of PHT*
Bakanay, 2005 [28]	Retrospective	US	226	16–68	Mortality
Belisario, 2016 [29]	Prospective cohort (2 cohorts)	Brazil	395	At time of stroke or end of f/u: 4.67; 9.35	Risk of stroke
			338	At time of high–risk TCD or end of f/u: 6.69; 8.68	High–risk TCD^†^
Bernaudin 2008 [30]	Prospective cohort	France	373	NR (children)	High TCD values
Bernaudin, 2015 [14]	Prospective cohort	France	189	Median age at first MRI/MRA: 5.4 Median age at MRI/MRA with cervical assessment: 8.7	SCI
Chaturvedi, 2018 [12]	Retrospective	US	150	Median: 25.5	Mortality
DeBaun, 2012 [31]	Cross-sectional	International	814	9.06 vs. 9.35	SCI
De Castro, 2008 [32]	Retrospective	US	125	39.3	Changes in TRV
Domingos, 2014 [33]	Prospective cohort	Brazil	261	NR	Stroke
Feld, 2015 [34]	Prospective cohort	US	247	36.2	Mortality
Gladwin, 2004 [35]	Prospective cohort	US	195	36	Association with TRV
Gurkan, 2010 [36]	Retrospective	US	40	5–20	Microalbuminuria
Hsu, 2003 [37]	Retrospective	US	314	2–16	TCD status
Iwalokun, 2012 [38]	Prospective cohort	Nigeria	103	20.7 vs. 15.7	Albuminuria
Kassim, 2015 [39]	Retrospective	US	430	≥21	Mortality
King, 2011 [40]	Prospective cohort	Jamaica	244	7.2	Microalbuminuria
King, 2014 [41]	RCT (2 cohorts)	US	150	9.2vs. 8.5	SCI
Knight-Madden, 2013 [42]	Prospective cohort	Jamaica	75	23.9 vs. 23.1	Mortality
Kwiatkowski, 2009 [43]	Retrospective	US	96	3.7	SCI
Kwiatkowski, 2011 [44]	Prospective cohort (2 cohorts)	US	195	10.9 vs. 7.6	Conversion to normal TCD
Lebensburger, 2019 [45]	Prospective cohort	US	91	5–21	Proteinuria
Lebensburger, 2011 [46]	Retrospective (2 cohorts)	US	144	NR	Microalbuminuria
Lobo, 2015 [47]	Prospective cohort	Brazil	125	27.6 vs. 34.7	TRV status
Makani, 2011 [48]	Prospective cohort	Tanzania	1,725	Median: 8	Mortality
Mawanda, 2011 [49]	Prospective cohort	Uganda	305	9.7	Microalbuminuria
McBurney, 2002 [16]	Retrospective	US	142	2–20	Microalbuminuria
McKie, 2007 [50]	Retrospective (2 cohorts)	US	191	3–20	Microalbuminuria/proteinuria
McPherson Yee, 2011 [51]	Retrospective (2 cohorts)	US	410	11.3	CKD and albuminuria
Naoman, 2010 [52]	Retrospective	US	105	Median: 37	TRV
Nebor, 2010 [53]	Prospective cohort	Guadalupe	189	34.8	Albuminuria
Nelson, 2007 [54]	Prospective cohort	US	53	12.1	Elevated TRV
Rankine-Mullings, 2015 [55]	Retrospective	Jamaica	40	13.9 vs. 15.3	Mortality
Sachdev, 2011 [56]	Prospective	US, UK	483	36	TRV
Sedrak, 2009 [57]	Prospective cohort	US	48	12	TRV
Silva, 2011 [58]	Prospective cohort	Brazil	291	6.2	Cerebrovascular disease
Sokunbi, 2017 [59]	Prospective cohort	Nigeria	175	8.8	TRV
Villagra, 2007 [60]	Prospective cohort	US	33	37 vs. 43	Elevated TRV
Voskaridou, 2007 [61]	Prospective cohort	Greece	84	35	TRV

CKD, chronic kidney disease; f/u, follow-up; MRA, magnetic resonance angiography; MRI, magnetic resonance imaging; NR, not reported; PHT, pulmonary hypertension; RCT, randomized controlled trial; SCI, silent cerebral infarct; TCD, transcranial Doppler; TRV, tricuspid regurgitant velocity; UK, United Kingdom; US, United States.

* PHT assessed by TRV and pulmonary artery systolic pressure measurement.

^†^ Defined as a time-averaged mean of the maximal velocity ≥200 cm/sec in the internal carotid or middle cerebral artery on two recordings.

**Fig 2:**
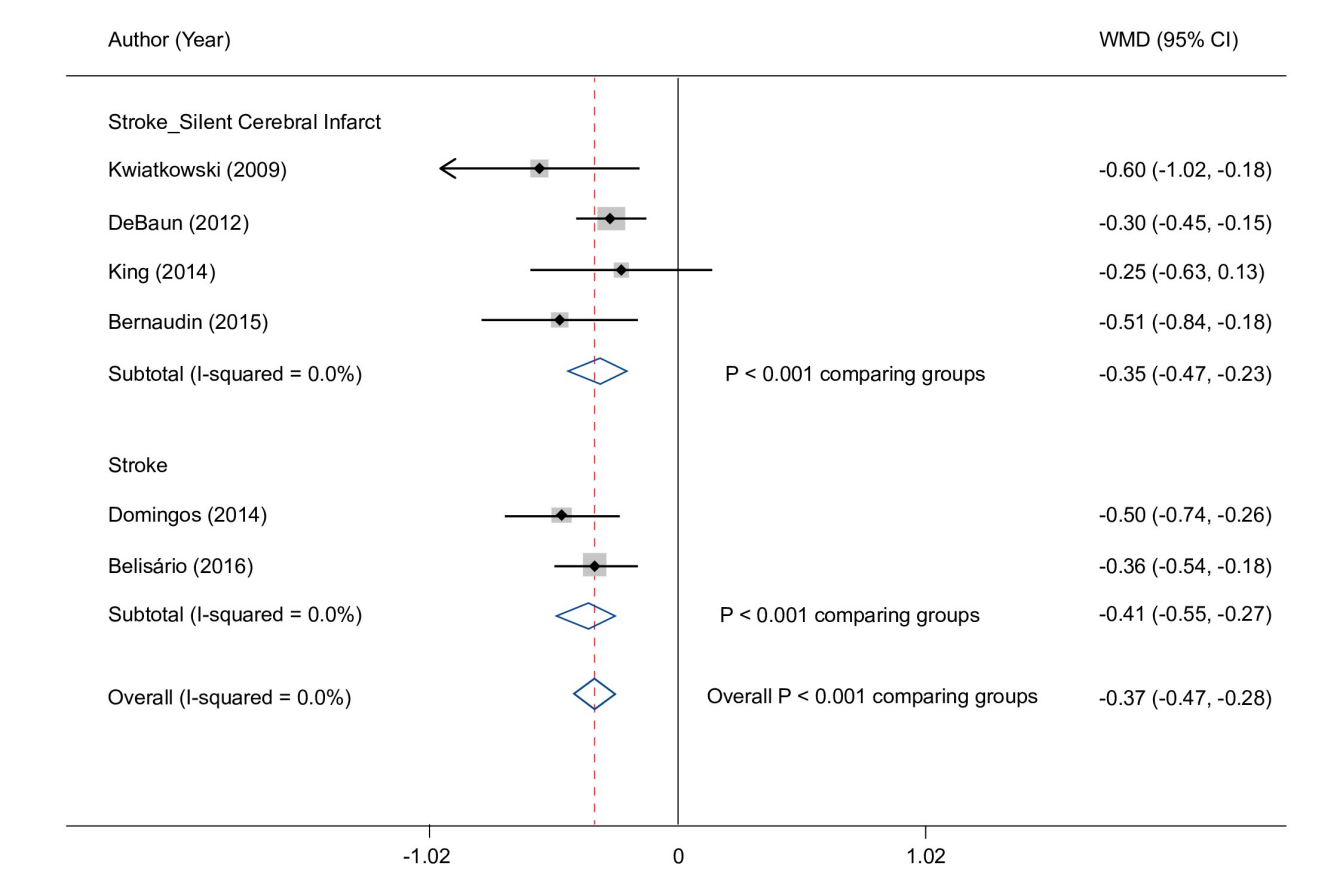
Hemoglobin difference in patients with stroke or silent cerebral infarct. * CI, confidence interval; WMD, weighted mean difference. *A sensitivity analysis was conducted with the study by King (2014) study removed and the meta-analysis results were essentially unchanged.

**Fig 3:**
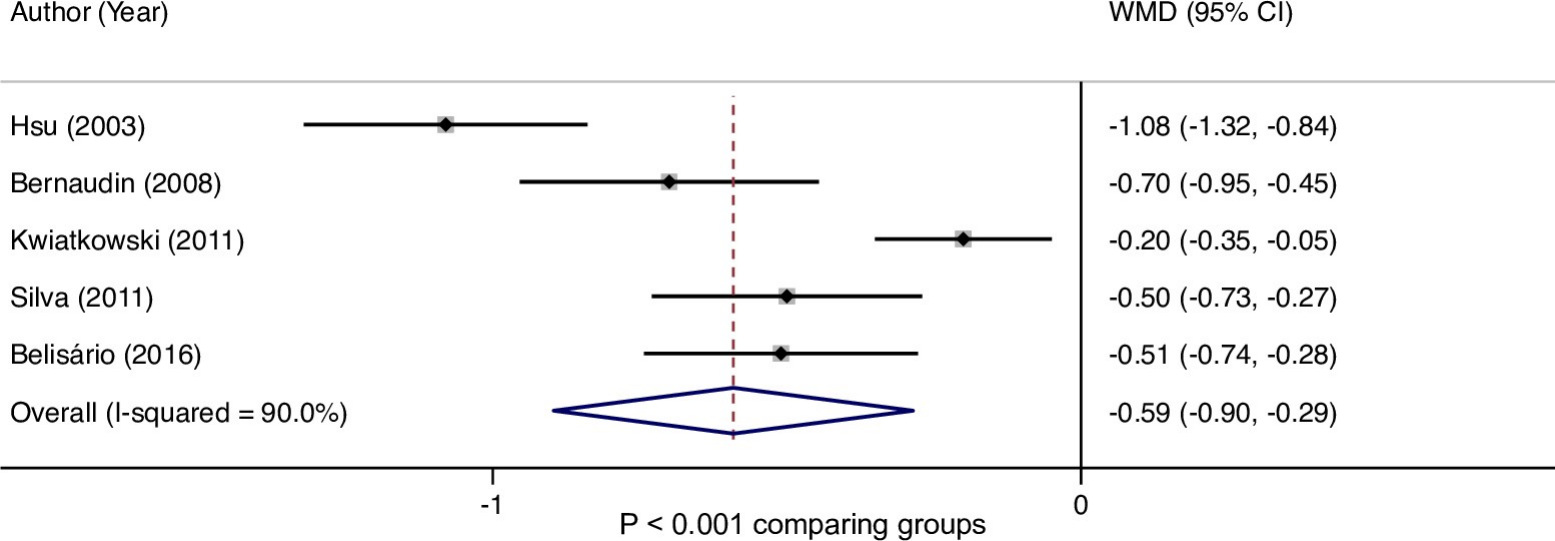
Hemoglobin difference in patients with TCD abnormalities. CI, confidence interval; TCD, transcranial Doppler; WMD, weighted mean difference.

Overall, the random effects meta–analysis showed that hemoglobin concentration was significantly lower overall by 0.4 g/dL (95% CI: 0.3, 0.5) in patients with a history of stroke (0.4 g/dL in patients with stroke/silent cerebral infarct and 0.4 g/dL in stroke alone) compared with those who had no history of stroke (Fig 2). In studies reporting transcranial Doppler (TCD) results, hemoglobin concentration was significantly lower by 0.6 g/dL (95% CI: 0.3, 0.9) in those with, compared to those without, an abnormal TCD velocity (Fig 3).

**Fig 4:**
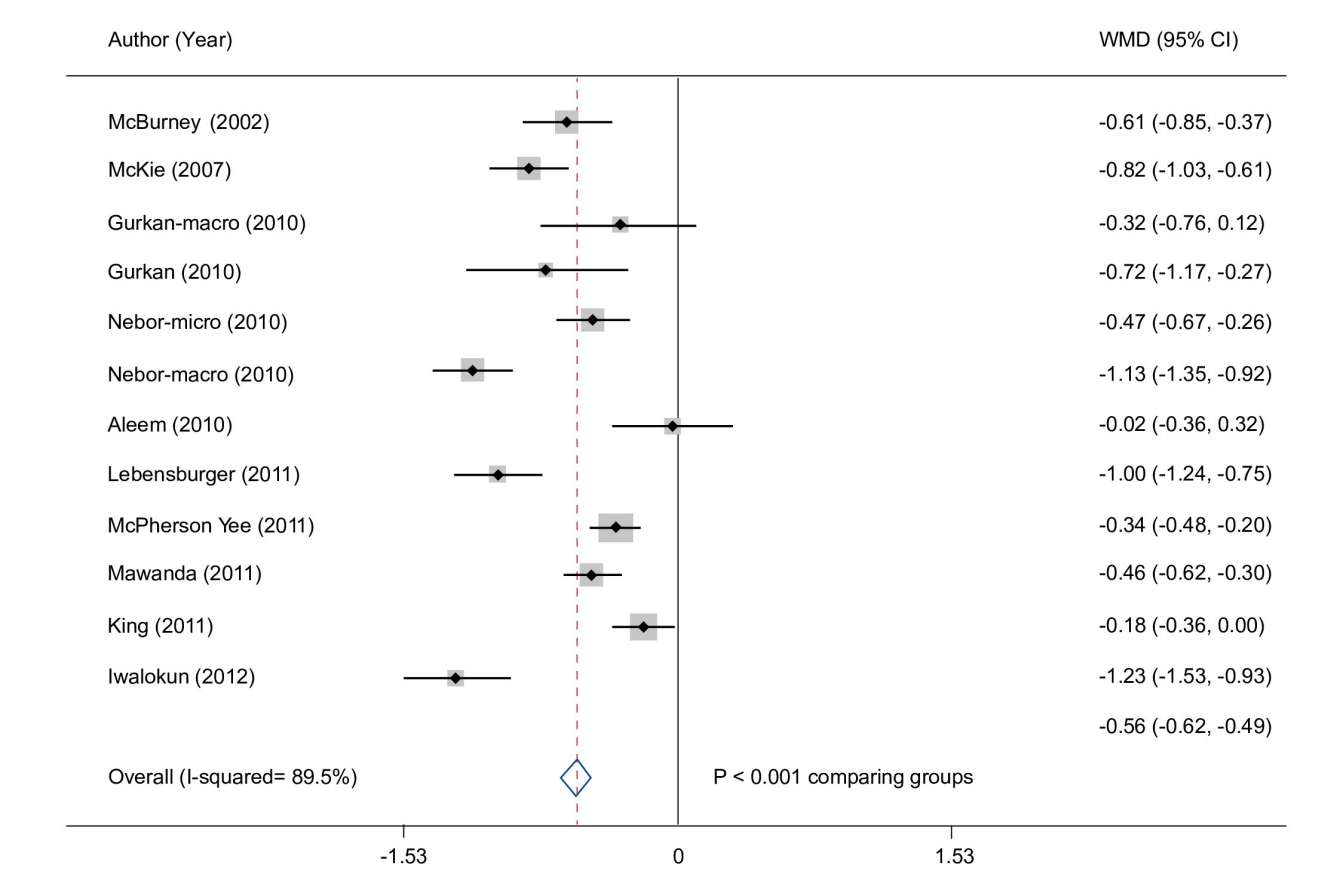
Hemoglobin difference in patients with albuminuria. CI, confidence interval; WMD, weighted mean difference.

The meta–analysis evaluating pediatric and adult patients showed a significantly lower hemoglobin concentration by 0.6 g/dL (95% CI: 0.5, 0.6) in patients with albuminuria (Fig 4). In a subgroup assessment of pediatric patients, individuals with moderately increased albuminuria had a significantly lower hemoglobin concentration by 0.7 g/dL compared with patients with normal albuminuria (95% CI: 0.5, 0.8) (S3 Appendix of S1 Fig).

**Fig 5:**
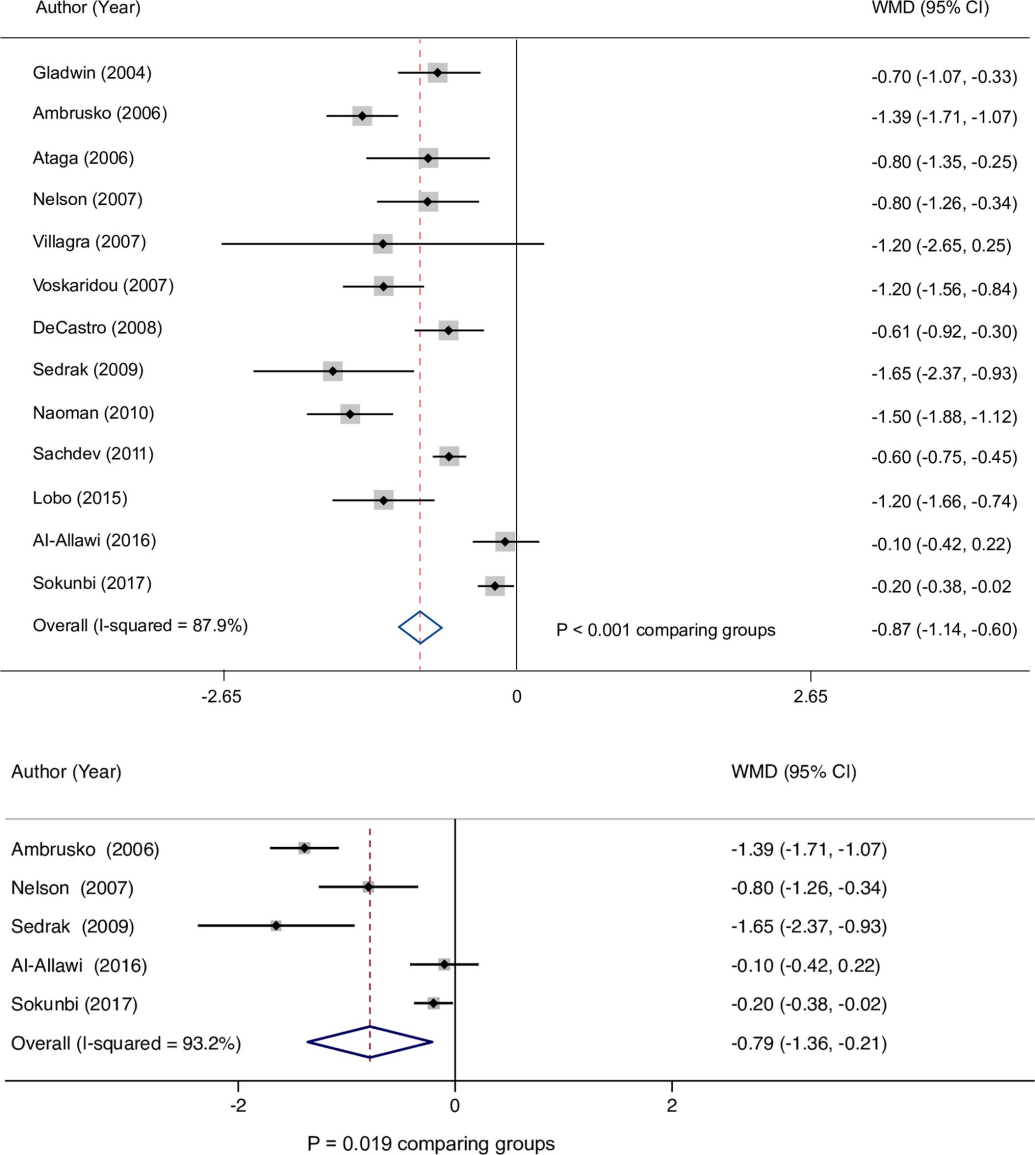
(A) Hemoglobin difference in patients with elevated estimated PASP. (B) Hemoglobin difference in pediatric patients with elevated estimated PASP. CI, confidence interval; PASP, pulmonary artery systolic pressure; WMD, weighted mean difference.

Among patients with an elevated estimated PASP, the meta–analysis showed hemoglobin concentration was significantly lower by 0.9 g/dL (95% CI: 0.6, 1.1) than in patients without an elevated estimated PASP (Fig 5A). Similar results were demonstrated among pediatric patients where hemoglobin concentrations averaged 0.8 g/dL (95% CI: 0.2, 1.4) lower in those with an elevated estimated PASP compared with patients without (Fig 5B).

**Fig 6:**
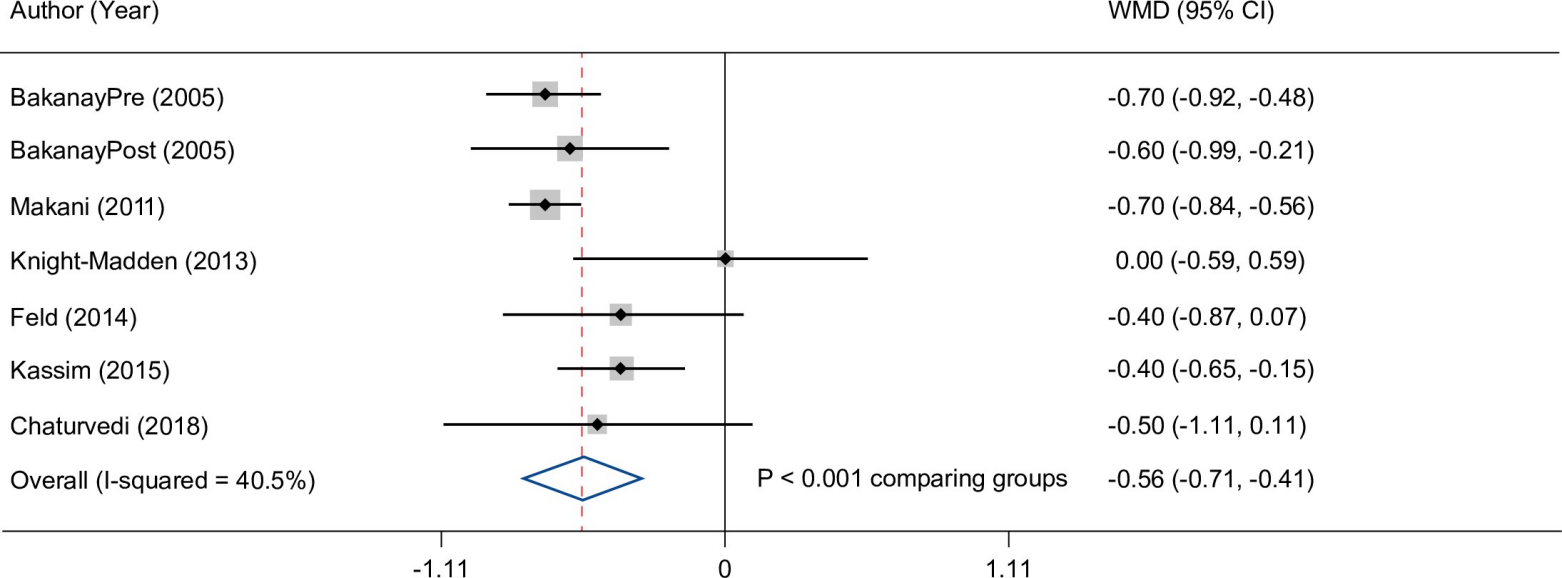
Hemoglobin difference in living and deceased patients. CI, confidence interval; WMD, weighted mean difference.

Overall, hemoglobin concentration was significantly lower by 0.6 g/dL (95% CI: 0.4, 0.7) in deceased versus living patients (Fig 6).

**Fig 7:**
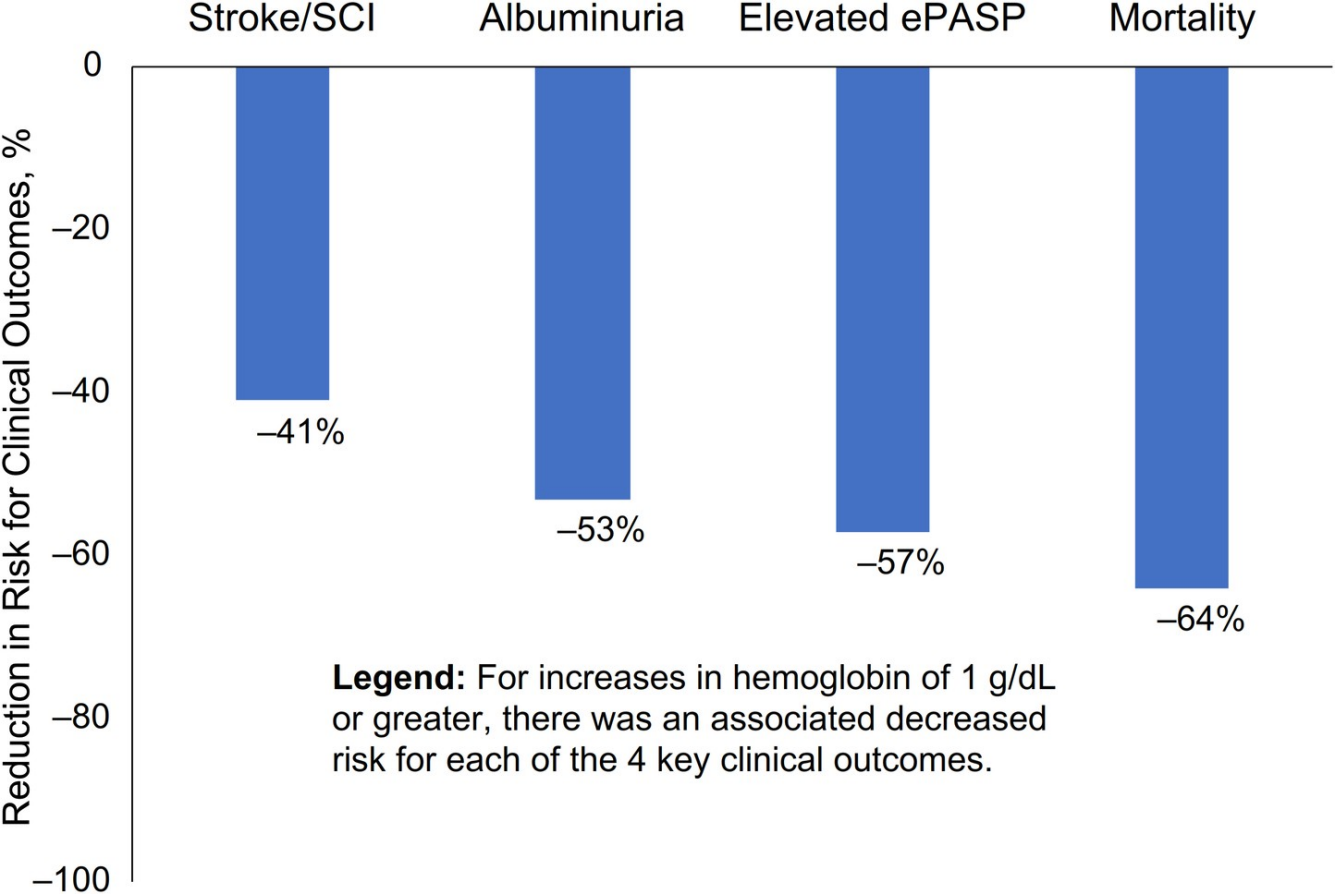
Modeled increase in hemoglobin and associated reduction in risk for negative clinical outcomes. CI, confidence interval; ePASP, estimated pulmonary artery systolic pressure; SCI, silent cerebral infarct.

Using data from the included literature, an additional meta–analysis was conducted to derive an estimation of potential reduction in risk for stroke/silent cerebral infarct, albuminuria, elevated estimated PASP, and mortality that might be associated with increases in hemoglobin concentration, Fig 7. Overall, the modeled risk reduction for negative clinical outcomes decreased at all modeled levels of increased hemoglobin concentration, most notably with improvements in hemoglobin concentration of 1 g/dL or greater. Specifically, this analysis demonstrated in a population with SCD that a hemoglobin concentration of ≥ 1.0 g/dL higher predicted a 41% reduction in the risk for stroke/silent cerebral infarct; the modeled risk reduction was 53% for albuminuria and 57% for elevated estimated PASP. An increase in hemoglobin of ≥ 1.0 g/dL in SCD patients was estimated to reduce the risk of mortality by 64%. Sensitivity analyses showed no studies influenced the outcomes using a jackknife analysis and the Begg and Egger statistics did not indicate any small sample bias.

## Discussion

This report focused on understanding the associations between hemoglobin concentration and clinical outcomes among children and adults with SCD. Among patients of all ages, lower hemoglobin concentration was consistently associated with higher incidence or history of stroke, silent cerebral infarcts, increased TCD velocity, albuminuria, elevated estimated PASP, and mortality. In aggregate, the differences in hemoglobin concentration between groups of individuals with and without a negative event were statistically significant across all clinical outcomes evaluated, highlighting the serious complications associated with anemia in the SCD patient population.

Our analyses are supported by data from seminal longitudinal studies, which provide evidence of the relationship between hemoglobin concentration and key clinical outcomes among patients with SCD. In an analysis of data from 3,764 patients from the Cooperative Study of Sickle Cell Disease (CSSCD), who ranged in age at enrollment from birth to 66 years, Platt et al demonstrated that patients with sickle cell anemia with hemoglobin below the 10^th^ percentile (≤ 7.1 g/dL) had a higher risk of death than all other patients (2.8 vs 1.1 deaths per 100 person–years) [9]. In a subsequent analysis of data from the CSSCD included in our review, the relationship between anemia and stroke was further elucidated [62]. The relative risk of infarctive stroke significantly increased by a factor of 1.85 per 1 g/dL decrease in hemoglobin concentration and the relative risk of hemorrhagic stroke increased by a factor of 1.61 per 1 g/dL decrease in hemoglobin concentration [62]. In the Jamaican Cohort Study of Sickle Cell Disease, in 17 of 310 children with homozygous SCD followed from birth in whom a stroke occurred, an acute decrease of hemoglobin concentration was a risk factor for stroke [63]. Additionally, Powars and colleagues described a single–center prospective cohort study of 725 patients with sickle cell anemia and 209 patients with sickle hemoglobin C disease in whom hypertension, proteinuria, and increasingly severe anemia predicted end–stage renal failure [10].

The present meta–analyses attempt to quantify the potential risk reduction that might be associated with increasing hemoglobin concentration in SCD patients. This analysis suggests that a hemoglobin increase of at least 1 g/dL might confer a 41% risk reduction for stroke and silent cerebral infarct. This prediction model is indirectly supported by evaluation of individuals with varying anemia severity based on SCD genotype. In patients with sickle cell anemia, co-existing alpha thalassemia yielded a relative risk reduction of 0.44 for stroke, with a multivariate analysis showing that the protective effect of alpha thalassemia is largely due to the improvement in hemoglobin concentration; alpha thalassemia was not a significant predictor of stroke after adjusting for hemoglobin concentration [62]. Further, the plausibility of the present findings is rational given the underlying pathophysiologic mechanism of ischemic cerebrovascular injury in SCD, where severity of anemia is associated with increased cerebral blood flow, decreased compensatory reserve, and a greater risk of stroke during periods of stress when the brain oxygen supply and demand are imbalanced [31].

The early onset of end–organ damage in SCD suggests the need for timely screening and appropriate intervention in children. Kwiatkowski et al noted that by 3.7 years of age, 27% of children with SCD had a silent cerebral infarct [43]. In addition, children with SCD suffer cognitive impairment, with low hematocrit being an independent predictor of intelligence scores and impaired cognitive functioning in patients with a history of stroke or silent stroke [64, 65]. Adults with SCD also show poorer performance on cognitive tests relative to controls, and anemia and increasing age is associated with lower neurocognitive performance [66]. The prevalence of albuminuria is up to 27% in children with SCD [36, 40, 51, 67, 68], with King and colleagues detecting this complication as early as 2.8 years of age [40]. Further, in a prospective cohort study in Minnesota, 31% of children over 10 years of age had evidence of elevated estimated PASP by Doppler echocardiography [54]. Although the investigators recommended screening children for pulmonary hypertension starting at age 10 [54], the clinical significance of elevated TRV in children remains uncertain.

The interventions available to increase hemoglobin concentration in individuals with SCD are limited. RBC transfusions, hydroxyurea, erythropoiesis stimulating agents and bone marrow transplants are therapeutic options with demonstrated effect. Increasing hemoglobin concentration via chronic RBC transfusions reduces stroke, silent cerebral infarcts, and abnormal TCD [14, 44, 69]. Hydroxyurea also reduced TCD velocity with an overall increase in hemoglobin concentration from baseline [70]. However, concerns regarding the long-term safety profile of these interventions suggest a need for additional therapeutic options to reduce anemia and hemolysis in SCD. The mechanism of hemoglobin concentration increase may be important. Raising hemoglobin concentration with an erythropoiesis stimulating agent may have a different clinical profile compared to increasing hemoglobin concentration with a therapy that reduces hemolysis. Voxelotor, a hemoglobin S polymerization inhibitor recently approved by the US FDA, demonstrated a sustained increase in hemoglobin levels and reduced hemolysis in patients with SCD [71, 72].

There are limitations to this study. Decisions regarding *a priori* inclusion/exclusion criteria, influenced by key research objectives, resulted in a rigorous selection process applied during the search, title/abstract, and full–text review phases. As a result, studies without specific qualifying information at a review phase were excluded, most notably during the screening of publication titles and abstracts. Additionally, few RCTs were identified for inclusion in the systematic review. Only 1 RCT met the *a priori* inclusion/exclusion criteria, evaluating outcomes in response to prophylactic blood transfusions [41]. Also, some individual patients may be represented in more than 1 cohort or publication, as several publications have arisen from landmark data sets. There was some heterogeneity between studies; high heterogeneity is often a realistic expectation for modeling real-world outcomes. Statistically we have controlled this variability by using random effects models to ensure that the overall reported effect size is valid and adjusted for within and between study variability. Beyond controlling for heterogeneity, we conducted analyses to identify possible sources of variability as reported in our sensitivity and subgroup analyses [73, 74]. Meta–analyses were not conducted for measures of hemolysis, as insufficient uniform data in analyzable form were identified from the studies that met inclusion criteria to inform the quantitative assessments. Lastly, the identified publications primarily evaluated the associations between hemoglobin concentration and clinical outcomes. While the consistency of findings across studies support the robustness of potential implications drawn from this data, we caution against the conflation of association and causation, and cannot explicitly state that raising the hemoglobin concentration will cause clinical improvement. Given the highly complex pathophysiology of SCD, addressing multiple factors such as RBC rheology, hemolysis, and vasculopathy warrants consideration. Further, controlled studies are required to confirm the effects of raising hemoglobin concentrations on clinical complications in patients with SCD.

In conclusion, comprehensive evaluation of peer–reviewed literature published over the last 20 years demonstrates a significant relationship between degree of anemia and worse clinical outcomes in individuals with SCD. While some heterogeneity existed among the studies, the pattern of lower hemoglobin concentration and higher risk of cerebrovascular disease, albuminuria, cardiopulmonary disease, and mortality was consistent for pediatric and adult patients with SCD. Meta–analyses further demonstrate that even relatively modest differences in hemoglobin concentration may be clinically meaningful. In aggregate, these results support that interventions to reduce anemia may confer clinical benefit in this patient population. Indeed, these results underscore the therapeutic importance of agents that increase hemoglobin and which have the potential to modify disease severity. Given that the multi–organ damage associated with hemolytic anemia begins at a young age, novel therapeutic options that can be used early in life are needed to interrupt the underlying pathogenic mechanisms of SCD.

## Supporting information

S1 Appendix Search strategies. (DOCX)

S2 Appendix Quality assessment. (DOCX)

S3 Appendix Meta-analysis. (DOCX)

S4 Appendix PRISMA checklist—Preferred reporting items for systematic reviews and meta-analyses checklist. (DOCX)
