# Supplementary material for: Low hemoglobin increases risk for cerebrovascular disease, kidney disease, pulmonary vasculopathy, and mortality in sickle cell disease: A systematic literature review and meta-analysis
Source: PLoS One. 2020 Apr 3;15(4):e0229959. doi: 10.1371/journal.pone.0229959 (PMC7122773; doi:10.1371/journal.pone.0229959)
Supplement: S1 Appendix — (DOCX) [file pone.0229959.s001.docx]

# Search Strategies

Table S1. MEDLINE Search Strategy. Ab abstract; MeSH, medical subject heading; pt, publication type; RBC, red blood cell; SCD, sickle cell disease; ti, title

| **Date of Search** | **February 26, 2019** |
| --- | --- |
| **Search** | **Query** |
| #1 | "Anemia, Sickle Cell"[Mesh] OR “sickle cell anemia”[tiab] OR “sickle cell anaemia”[tiab] OR “sickle cell disease”[tiab] OR SCD[tiab] |
| #2 | Efficacy[tiab] OR safety[tiab] OR effectiveness[tiab] OR “adverse event”[tiab] OR tolerability[tiab] OR “adverse effect”[tiab] OR mortality[tiab] OR death[tiab] OR  randomized[tiab] OR randomized[tiab] OR placebo[tiab] OR controlled[tiab] OR blind*[tiab] OR trial[tiab] OR allocat*[tiab] OR assign*[tiab] OR “real world”[tiab] OR “real-world”[tiab] OR observational[tiab] OR retrospective[tiab] OR prospective[tiab] OR “cross sectional”[tiab] OR registry[tiab] OR database[tiab] OR cohort[tiab] |
| #3 | #1 AND #2 |
| #4 | Hemoglobin[tiab] OR haemoglobin[tiab] OR (organ[tiab] AND damage[tiab]) OR reticulocyte[tiab] OR bilirubin[tiab] OR “lactate dehydrogenase”[tiab] OR hemolysis[tiab] OR haemolysis[tiab] OR (dense[tiab] AND (“red blood cell*”[tiab] OR RBC*[tiab])) |
| #5 | #3 AND #4 |
| #6 | #5 NOT (animals[MeSH] NOT humans[MeSH]) |
| #7 | #6 NOT (review[PT] NOT (systematic OR “meta analysis”)) |
| #8 | #7 NOT (case reports [pt] OR editorial [pt] OR letter [pt] OR comment [pt]) |
| #9 | #8 Filters: Publication date from 1998/01/01 to 2017/08/29; English |

Table S2. EMBASE Search Strategy. ab, abstract; exp, explode; py, publication years; RBC, red blood cell; SCD, sickle cell disease; ti, title

| **Date of Search** | **February 26, 2019** |
| --- | --- |
| **Search** | **Query** |
| #1 | 'sickle cell anemia'/exp OR 'sickle cell anemia':ab,ti OR 'sickle cell anaemia':ab,ti OR 'sickle cell disease':ab,ti OR scd:ab,ti |
| #2 | efficacy:ab,ti OR effectiveness:ab,ti OR safety:ab,ti OR 'adverse event':ab,ti OR 'adverse effect':ab,ti OR tolerability:ab,ti OR mortality:ab,ti OR death:ab,ti OR  randomized:ab,ti OR randomised:ab,ti OR placebo:ab,ti OR controlled:ab,ti OR blind*:ab,ti OR trial:ab,ti OR allocat*:ab,ti OR assign*:ab,ti OR 'real world':ab,ti OR observational:ab,ti OR retrospective:ab,ti OR prospective:ab,ti OR 'cross sectional':ab,ti OR registry:ab,ti OR database:ab,ti OR cohort:ab,ti |
| #3 | 1 AND 2 |
| #4 | hemoglobin:ab,ti OR haemoglobin:ab,ti OR reticulocyte:ab,ti OR bilirubin:ab,ti OR 'lactate dehydrogenase':ab,ti OR hemolysis:ab,ti OR haemolysis:ab,ti OR (organ:ab,ti AND damage:ab,ti) OR [dense:ab,ti AND ('red blood cell*':ab,ti OR rbc*:ab,ti)] |
| #5 | 3 AND 4 |
| #6 | Limits: English, Humans, 1998-2017(py) |
| #7 | Limits: Publication types-Articles, Articles in Press, Reviews |
