# Supplementary material for: Low hemoglobin increases risk for cerebrovascular disease, kidney disease, pulmonary vasculopathy, and mortality in sickle cell disease: A systematic literature review and meta-analysis
Source: PLoS One. 2020 Apr 3;15(4):e0229959. doi: 10.1371/journal.pone.0229959 (PMC7122773; doi:10.1371/journal.pone.0229959)
Supplement: S2 Appendix — (DOCX) [file pone.0229959.s002.docx]

# 2.0.Quality assessment

Overall, prospective studies ranged in scores of 4 to 7, with a mean score of 6 and a median score of 6. The maximum score on the Newcastle-Ottawa Scale is 8, suggesting that studies approaching this score have fewer potential sources of bias comparatively. While patient populations ranged from being somewhat to truly representative of community sickle cell disease patients, those that evaluated 2 cohorts generally relied on a population drawn from the same community. Several included studies were cross-sectional in nature (and were not downgraded with respect to quality for the design), and the majority of analyses relied on secure medical records or structured interviews as primary data sources. Potential sources of bias included highly selected patient populations, presence of the outcome of interest at baseline, and a paucity of data describing patient follow-up.

Retrospective analyses were evaluated across domains, including the data source, discussion of study design and limitations, patient populations (e.g., eligibility criteria, baseline characteristics), provision of primary outcome assessments, and authors’ conclusions. Potential sources of bias included insufficient detail about the data source and related validity. Further, several included analyses failed to provide adequate data regarding included subjects and neglected to discuss limitations in the research designs and analyses.

One RCT (King 2014) was included, and overall, it provided insufficient data to allow evaluation of sources of bias.
