# Supplementary material for: Low hemoglobin increases risk for cerebrovascular disease, kidney disease, pulmonary vasculopathy, and mortality in sickle cell disease: A systematic literature review and meta-analysis
Source: PLoS One. 2020 Apr 3;15(4):e0229959. doi: 10.1371/journal.pone.0229959 (PMC7122773; doi:10.1371/journal.pone.0229959)

# 3.0. Meta-analysis

**Fig S1** **Comparison of hemoglobin between pediatric patients with and without moderately increased albuminuria.** CI, confidence interval; WMD, weighted mean difference.


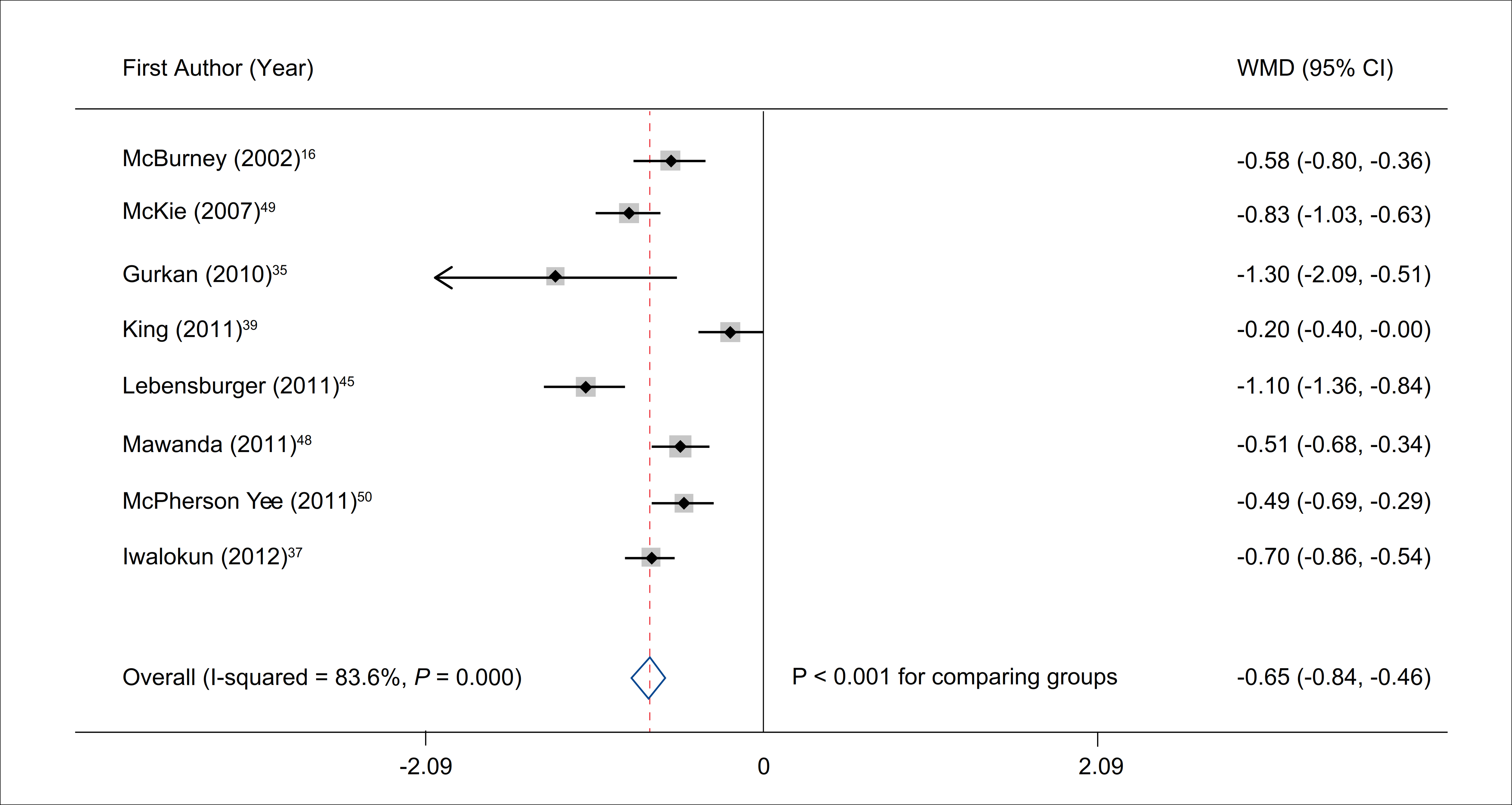

Supplement: S3 Appendix — (DOCX) [file pone.0229959.s003.docx]
